# Self-Organizing IoT Device-Based Smart Diagnosing Assistance System for Activities of Daily Living

**DOI:** 10.3390/s21030785

**Published:** 2021-01-25

**Authors:** Yu Jin Park, Seol Young Jung, Tae Yong Son, Soon Ju Kang

**Affiliations:** 1 Center of Self-Organizing Software, Kyungpook National University, 80 Daehakro, Bukgu, Daegu 41566, Korea; ilbsyjp@gmail.com (Y.J.P.); pipikako@gmail.com (T.Y.S.); 2 School of Electronics Engineering, College of IT Engineering, Kyungpook National University, 80 Daehakro, Bukgu, Daegu 41566, Korea; snowflower@ee.knu.ac.kr

**Keywords:** activity of daily living, IoT device, home healthcare

## Abstract

Activity of daily living (ADL) is a criterion for evaluating the performance ability of daily life by recognizing various activity events occurring in real life. However, most of the data necessary for ADL evaluation are collected only through observation and questionnaire by the patient or the patient’s caregiver. Recently, Internet of Things (IoT) device studies using various environmental sensors are being used for ADL collection and analysis. In this paper, we propose an IoT Device Platform for ADL capability measurement. Wearable devices and stationary devices recognize activity events in real environments and perform user identification through various sensors. The user’s ADL data are sent to the network hub for analysis. The proposed IoT platform devices support many sensor devices such as acceleration, flame, temperature, and humidity in order to recognize various activities in real life. In addition, in this paper, using the implemented platform, ADL measurement test was performed on hospital patients. Through this test, the accuracy and reliability of the platform are analyzed.

## 1. Introduction

In the international community, there has been an increase in the social problems affecting the elderly population. To improve the quality of life of this growing elderly population, various approaches are being considered with respect to not only basic housing, medical care, and dietary lifestyles but also social activities and welfare. Among these, the medical field is concerned with research on the prevention and treatment of brain diseases, such as dementia, which are fatal in the elderly. Brain disease is diagnosed in the hospitals using various methods; however, when it is medically performed, expensive tests such as brain scans are required. Hence, doctors usually diagnose brain disease through interviews with the patients or their caregivers using questionnaires [1]. However, this method is prone to errors because the amount of information obtained about the patient’s life is less in this approach, and the tests themselves are performed through conversations or temporary assessments. IoT sensor devices are also used to diagnose patients with brain diseases. The decline in living ability in patients with brain disease has a close relationship with the decrease in physical exercise ability. In order to directly test the patient’s motor skills, a gait test using acceleration sensors, wearable device and foot pressure pads is being conducted by medical researchers. This gait test is used as an indicator of brain disease diagnosis along with an interview and to determine the patient’s motor function decline [2,3]. Gait testing based on an acceleration sensor is used as an analysis method that detects patient’s gait abnormality with high accuracy. Gait analysis conducted in a hospital is a basic test method that helps diagnose Parkinson’s disease with the patient’s motor ability. Recently, studies are being conducted to detect abnormal walking of patients using data processing of sensor data, waveform analysis, and deep learning technology [4,5]. Furthermore, gait test research is applied to real-time data collection and gait abnormality analysis in patient life through smartphones and wearable devices. However, the ability to use tools and cognitive functions, which can be seen as the patient’s life ability, are very abstract, and long-term analysis is required to obtain effective information.

In this study, to improve the prevention and diagnosis of brain diseases in the elderly, such as mild cognitive impairment (MCI) and dementia, we investigated a method to replace and assist the existing diagnostic tests, with cooperation from medical experts. Currently, the criteria used in the diagnostic tests are mainly analyzed using activities of daily living (ADL) [6]. The ADL consists of various actions in daily life, such as moving, eating, sleeping, washing, cooking, and using home appliances [7]. For the diagnosis of brain disease, it is important to determine the ADL activity patterns and occurrence of unnecessary or abnormal behaviors in the patient’s life. However, these routine behavioral observations require long test periods for better accuracy and reliability; further, they may also be prone to observer errors. Herein, we propose a diagnostic system for the actual ADL measurement using an IoT sensor device and sensor network. The proposed ADL measurement system comprises a resource device (RD), a mobile identification device (MID), and a location anchor node (LAN). The RD is connected to an external sensor or home appliance to detect a user’s ADL event, which is then autonomously recognized by the RD from the surrounding MID to detect the user who generated the corresponding ADL through self-organizing M2M (Machine to Machine) communication between the two devices. For communication between the RD and MID, we propose a method of interworking with low frequency (LF) and Bluetooth low energy (BLE) communication. By integrating these two modes of communication, faster and more accurate user recognition is achieved, and the system compatibility is improved using a data communication method through a smartphone or sensor network. The ADL event information detected through the RD is transmitted to the LAN and then to the server for event storage and analysis. In addition, the LAN configures a location-based management system that determines and manages the location information of the RD and MID with the received signal strength indicator (RSSI) of a radio frequency (RF) signal. This location recognition method is mainly used in RSSI analysis techniques through Wifi or magnetic fields in indoor-navigation-related research [8,9,10]. In this work, we use unit space-based (such as room, communication range) location recognition through information exchange via the LAN for fast location recognition [11].

To verify the diagnosis system proposed in this paper, individual diagnosis tests were conducted for hospital patients. The pilot test was performed by classifying the patients into groups as Normal/MCI/Dementia to confirm the cognitive ability and accuracy of the system. The future development of an ADL diagnostic system using the method proposed in this paper aims to not only replace the diagnostic tests in hospitals but also collect ADL events of users over long durations through home installations. The remainder of this paper is structured as follows. Section 2 describes the ADL test and related research on smart home-based technologies, and Section 3 explains the overall configuration of the proposed system. Section 4 presents the detailed design and communication protocol of each device used, and their implementation and performance evaluation are presented in Section 5. Finally, Section 6 presents a discussion of the direction of future research with the developed platform.

## 2. Related Research

### 2.1. Activities of Daily Living

ADL ability in elderly living is an important factor determining the quality of life [12]. The ADL ability assessment is thus important for predicting and treating brain diseases, such as cognitive impairment and dementia [13,14,15,16,17]. In the field of IoT related research, studies on behavioral perception using various sensors for ADL evaluation, base communication networks, and service applications are being actively conducted. In the case of wearable sensor devices, such as smart bands, studies are being conducted to recognize the basic behaviors of users, such as walking, running, and standing, through integrated acceleration sensors [18,19]. Motion recognition technologies use acceleration sensors and analyze their results for each part of the user’s body. This technology is used not only in sports for correcting the athlete’s posture but also in ADL measurements to discriminate walking, running, standing, and sleeping with abstract behavioral recognition. The fall detection utility in wearable devices can be applied to actual fields for user safety by recognizing a user’s emergency situation and notifying their caregiver [20]. Research on wearable devices is thus helping improve user health and safety, including monitoring through ADL as well as vital measurements [21]. However, the practical use of such wearable sensor devices must satisfy the requirements of low-power and real-time operation as well as adequate usability of the device [22,23].

### 2.2. Ambient Assist Living and Smart Home Technology

Sensor technologies for ADL measurements use various environmental sensors, such as temperature/humidity/motion/color sensors [24], which are widely used in SMART home technologies; further, they are being studied in sensor fusion and big data analyses. SMART home and sensor data analysis technologies are being developed through ambient assist living (AAL) goals to assist in the daily lives of the elderly and as tools to realize active aging [25,26,27,28,29]. ADL analysis in SMART homes require data collection of user activity through environmental sensors installed in homes. The collected data are classified and filtered by considering the different properties between the sensors before performing behavioral analysis. Using sensor data processing research, more accurate and highly responsive analyses can be performed [30,31]. Behavior recognition for ADL evaluation uses various analysis methods and sensor equipment to cover numerous situations that occur in daily life. Lee, Y-S. et al. studied a home healthcare system that detects abnormal events such as falls through vision-based image analysis [32]. Recently, vision technologies have been in the spotlight with the advancement of big data and machine learning as well as the use of more accurate and complex analyses that could not be performed earlier. Chung, K. et al. proposed a noncontact sleep stage detection using a sound sensor and studied a polysomnography method that can be easily used in SMART homes rather than hospitals [33].

IADL, an instrumental activity analysis method, is one of the important elements used in the analysis of brain diseases in the elderly through ADL [34]. In particular, the usage of everyday home appliance records such as TVs, refrigerators, and coffee pots, can be easily collected, and these data have high value for analyzing user behavior patterns. Abdulsalam Yassine et al. collected daily life home appliance usage data through smart meters and used them as big data to analyze user behavior patterns [35]. In addition, they suggested its use in the healthcare field to detect abnormal behavioral conditions of the elderly to analyze behavior patterns and notify their guardians. Similarly, Kazuki Moriya et al. used the ECHONET lite appliance and motion sensor to directly collect On/Off usage records and user position information from home appliances [36]. They used the collected information as samples for machine learning. Tan T.-H. et al. proposed an algorithm for classifying oblivious and abnormal events in elderly life in SMART homes through analysis of the ADL events [37]. For ADL analysis and utilization of SMART home technology, the sensor communication network used for data collection and processing is also an important field of research, in addition to sensor and analysis technologies. Hemant Ghayvat et al. defined Zigbee and various wireless communication protocols to study real-time streaming services for ADL service utilization [38]. To improve the SMART home technology to a practical service level, a communication network and technology that guarantees real-time utility is essential.

## 3. Test Scenario and Proposed System Architecture

### 3.1. Scenario for ADL Test

**Figure 1:**
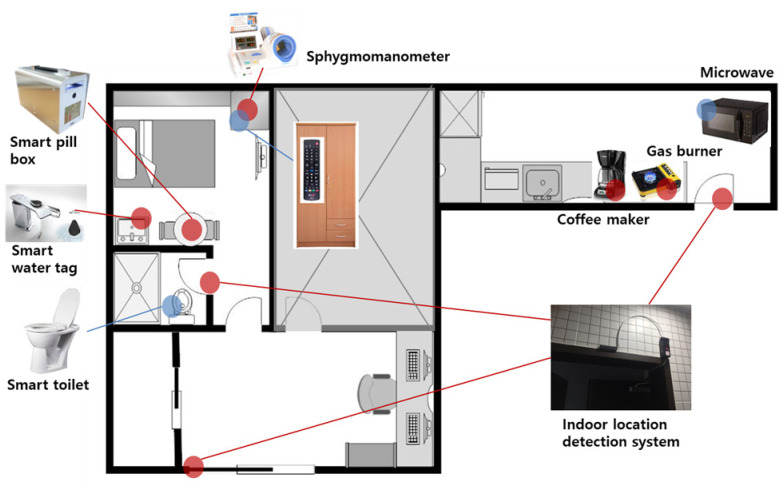
Activity of Daily Living (ADL) Device Layout for Pilot Test.

The tests of the ADL diagnostic system presented herein were performed on patients visiting the hospital. The tests were conducted in elderly patients over 60 years of age (38 person, 23 men, 11 women), and each patient was classified into one of three groups: Normal/MCI/Dementia. The test patient group was classified into 15 normal patients, 11 persons with mild cognitive impairment (MCI), and 12 persons with dementia. For testing of the ADL diagnosis system, the proposed RD and LAN were installed in the test place along with the equipment. The patients wore smart bands on their wrists during the entire test period. Figure 1 shows the arrangement of the devices used in this test. Each test was performed by focusing on the patient’s mobility and ability to use the tools/equipment. To detect the patient’s mobility, an access detection system using LF communication was installed at each entrance of each of the test sites. The entrance detection system was installed at the entrances of Test Room 1, Test Room 2, and the bathroom to measure the movements of users and the travel times between each pair of points. In each test room, the RD for measuring tool usage ability was installed for each equipment. The equipment installed in Test Room 1 is a medication box, a blood pressure monitor, a toilet, and a water tag. This setup was used to measure toilet usage behavior and ability to use medical devices. In Test Room 2, a coffee pot, gas burner, and microwave oven were placed to measure cooking behaviors performed in the kitchen. Each device has an RD to measure the patient’s ADL operation status. In addition, a LAN was installed in each test room for broadcasting, control during the ADL test, and analysis of ADL result data. For each test, the RD recognizes the patient’s ADL and delivers the result to the LAN. Details on the functions and operations of the RD and LAN are covered in Section 4.

First, when the patient arrives at the test site, the test manager creates a patient consent for the test. The test manager additionally explains the overall test motive, test progress, and basic test tips. Thereafter, before starting the test, the test manager sends the test start to the LAN by pressing the button of the RD on the wall of Test Room 1, the test starting place. When the test starts in this way, the LAN sequentially sends test broadcasts to convey the test instructions to the patient. The patient listens to the broadcast and performs the operation indicated by the broadcast, and the results are measured through the RD. The measured results are transmitted to the LAN to record the success of the test, and the following test instructions are broadcast according to the results. During the test process, LAN records not only the success or failure of the test but also the test execution time and log information of all events occurring between the tests. ADL tests that are sequentially conducted from Test Room 1 to Test Room 2 are performed in the same order as in Table 1.

The entire test consists of general behaviors in the daily lives of elderly patients. In Test Room 1, at this time, to measure the patient’s mobility, the time required for the patient to find and move to Test Room 2 and the success of the task are measured through the access system. Further, the patient’s use of the toilet and sink and their ability to monitor their blood pressure and take medication are tested periodically. After the primary test in Test Room 1, the patients move to Test Room 2, which is set up as a kitchen environment. At this time, to measure the patient’s mobility, the time required for the patient to find and move to Test Room 2 and the success of the task are measured through the entrance detection system. In Test Room 2, the ADL test is performed under the same environment as in a kitchen. The LAN instructs the user to operate the gas stove, coffee pot, and microwave through broadcasts to collect the patient’s normal operation of the device and successful execution status within the time limit. Lastly, the LAN instructs the user to move to Test Room 1, where the user first started, and measures their mobility. The test manager ends the entire test by pressing the test end button of the RD. After the test is completed, the ADL data and log information stored in the LAN are recorded as test results for each patient.

### 3.2. Smart ADL Diagnosing Assistance System

The smart ADL diagnosis assistance system proposed in this work recognizes the user’s ADL through the RD and collects and analyzes the results through the LAN. The entire test is controlled through vocal broadcasting via the LAN. The LAN analyzes and classifies the ADL results based on the time when the start broadcast for each test is completed. Thus, the LAN can be used to record and analyze the test results and prompt the next test or resume the test through re-broadcasting. The ADL results reported by the LAN are analyzed and controlled through the E-coaching system implemented through CLIP, a rule-based system [39]. When the ADL results are reported or the test event time is complete, the E-coaching system analyzes the results by triggering a rule with the event and commences the test accordingly. In this manner, all ADL tests are automated, and the results are derived by analyzing the patient’s ADL ability.

## 4. Detailed Design

### 4.1. Location System

**Figure 2:**
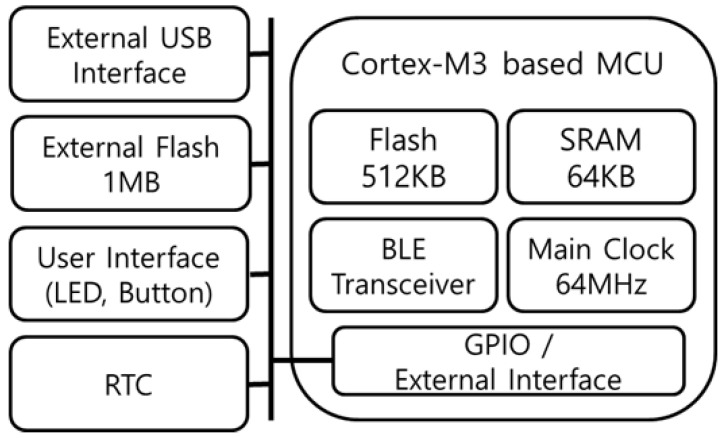
Hardware Block Diagram of Location Anchor Node.

The LAN manages the devices in its own cell area by analyzing the RSSI of the Bluetooth advertising beacon from the RD and MID. The RD and MID location information in the cell collected through this method is transmitted to the connected hub device (HD) through a USB interface. The HD is a Linux-based development board that executes the E-coaching system and location management system. Thus, the LAN is designed to embed not only the Bluetooth communication function but also the computing power and USB interface functions for data processing. Figure 2 represents the hardware block diagram of the LAN. 

The RD and MID always perform Bluetooth advertisement at a fixed period. Each BLE advertising data includes the 4-byte unique ID of the device, current device protocol status, and handle information to access the BLE profile. This unique ID comprises 3-byte device ID and 1-byte service ID to distinguishes the device characteristics. The RD and MID have ID systems in the same format, so the identification of a device as a mobile tag device or various types of RD services is achieved through the service ID. Table 2 specifies the service ID and type information of the ADL events. 

The communication protocol states of the RD and MID are divided into idle and identification states. In the idle state, the LAN sends the corresponding advertising beacon signal to the HD, including only the basic service ID and BLE profile information, to determine whether the device exists in the cell. The identification state is used for self-organized M2M communication between the RD and MID when an ADL event occurs. When an ADL event occurs, the RD sends an LF signal to the surroundings; the MID includes the RSSI value of the LF signal received from the RD in the identification state in the BLE advertising data and transmits the location information using the LF signal to the RD. The LF signal is in the 125 kHz band and has a large wavelength. Thus, RSSI having high transmittance of the LF signal enables accurate position recognition with error in the order of centimeters. The M2M communication scheme proposed in this paper is described in the next section. Table 3 shows the format of the BLE advertising data.

The LAN periodically scans the BLE advertising data and collects information on the peripheral devices. The collected MID and RD BLE advertising data are then transmitted to the HD connected via USB; using this information, the HD manages the device. Since the RD needs to transmit the ADL results to the HD through LAN, additional protocols are required. When the cell unit location information is determined through the RD’s BLE advertising analysis, the LAN performs a location registration process through the BLE connection with the RD in its cell. Through this location registration process, the BLE connection between the LAN and RD is always maintained, and immediate reporting is possible when an ADL event occurs. In addition, by constantly checking the connection status of the RD, it is possible to solve any communication problems and retransmit data so that more stable ADL events can be collected.

### 4.2. Resource Device

**Figure 3:**
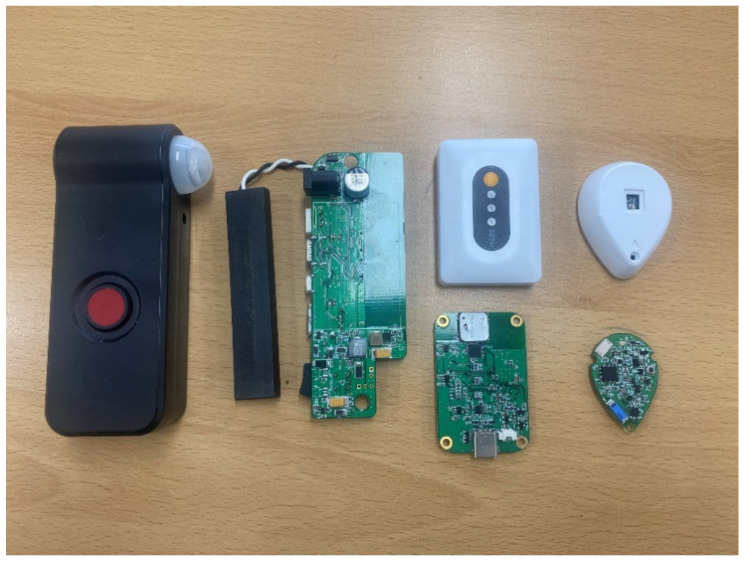
Implementation of Resource Device.

The RD recognizes user activity through various methods and delivers the collected activity information to the LAN. The RD device is using the platform we developed for this test. To implement RD, we designed the hardware by configuring the sensor, MCU and communication interface, and produced the development board. In addition, we have developed our own Real-Time Operating System (RTOS), communication protocols and programs built into the RD’s software. Figure 3 shows the implemented RD devices. The RD is divided into fixed-type stationary resource device (SRD) and sensor-type external sensor device (ESD). The SRD is primarily connected to home appliances, such as gas stoves, coffee pots, and blood pressure monitors, that are frequently used. The SRD recognizes the use event or result of use of the home appliance through wired communication such as UART and I2C, I/O interrupt, and analog sensor input. The SRD uses a 125 kHz LF communication method for user recognition of the home appliances and BLE communication for data transmission/reception. Activity events collected through the SRD are transmitted to the LAN that manages the location of the device. In addition, the SRD’s LF communication function is utilized as an entrance recognition system. This part will be discussed later.

The ADL event includes not only equipment usage history through the SRD but also user behaviors through the environmental sensor of the ESD. As an example, the ESD has a built-in acceleration sensor to check the usage of the faucet and recognize whether the user is using water as an activity event. The ESD does not include LF communication and uses only the BLE. Depending on the environment and application, the ESD is smaller in size and requires lower power, so it is designed as a device with fewer resources than the SRD. Similar to the SRD, the ESD is registered in the local LAN and transmits the user event results.

#### 4.2.1. Stationary Resource Device

**Figure 4:**
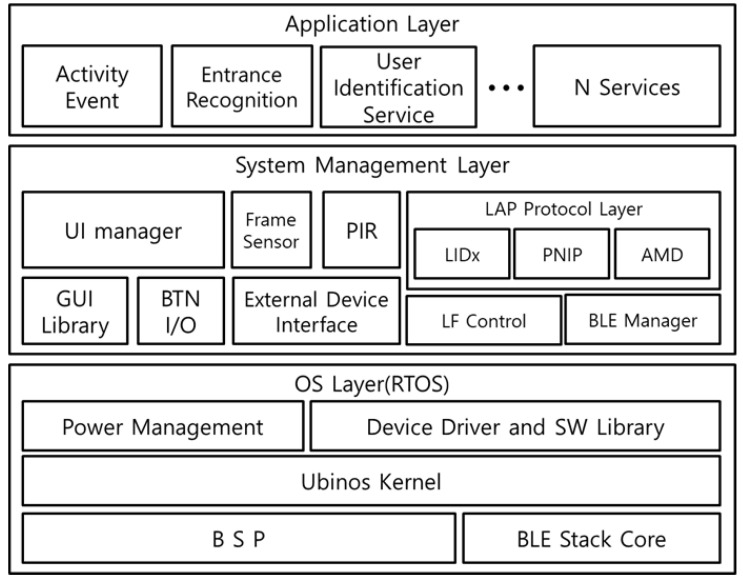
Software Architecture of Stationary Resource Device.

Figure 4 shows the software structure of the SRD. The Operating System(OS) layer consists of a BSP-device driver for hardware device control and includes the power management and Bluetooth stack core for low power operation. The system management layer is divided into the external device interface for user equipment connection and protocol layer for communication between the external devices using the LF-BLE communication. The external device interface collects the activity event information from various user equipment through I2C, SPI, UART, I/O interrupt, and sensor inputs. The protocol layer is responsible for user identification using the LF-BLE and communication with the LAN and ESD. The SRD provides various services for user activity event reporting through the functions of these two system layers.

#### 4.2.2. Mobile Identification Device

**Figure 5:**
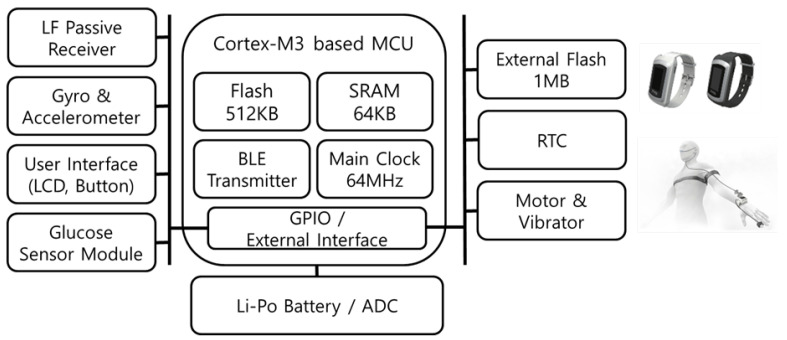
Hardware Block Diagram of Mobile Identification Device.

In the activity event report protocol of the smart diagnosing system proposed herein, the user of the corresponding activity event is recognized through LF communication between the SRD and MID [40]. The MID is designed in the form of a smart band or mobile tag that the users can wear, as shown in Figure 5, and provides user identification of the activity event through the LF passive receiver and BLE communication function. The MID receives the LF signal from the SRD and is ready for operation. The MID is a mobile device with limited resources, so it efficiently manages power consumed for communication [41]. In addition, the MID estimates the distance to the SRD through the received LF RSSI value; the estimated distance is included in the BLE advertising data and transmitted to the SRD.

#### 4.2.3. ADL Activity Report

**Figure 6:**
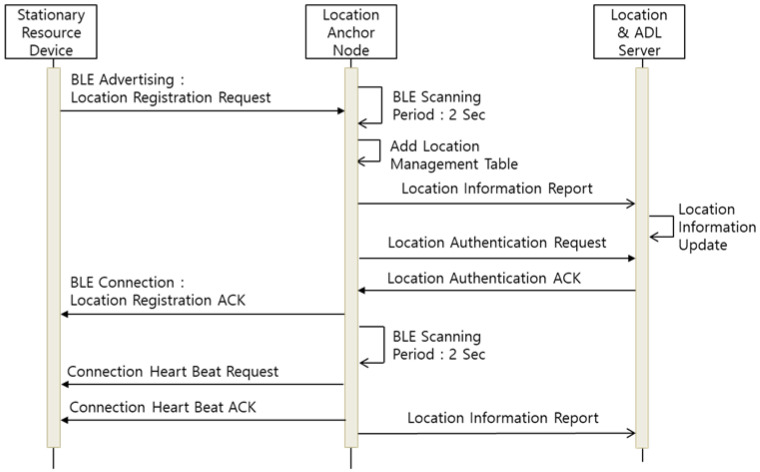
Cell Management and Location Registration Protocol.

The ADL event is collected through the self-organized M2M communication protocol of the MID–SRD–LAN. First, prior to operation of the entire communication protocol, the SRD and MID are registered in the LAN that manages the corresponding cell area. Figure 6 represents the location registration process between the SRD and LAN. 

The SRD transmits its ID and location registration request to the nearest LAN through BLE advertising. The LAN receiving the SRD’s request checks whether the corresponding SRD exists in its cell through the location server, which estimates the location information of the RD and MID by collecting the BLE beacon information received from the LAN. The SRD registered through the location information is managed through the LAN’s location management table. Thereafter, the SRD maintains the BLE connection with the LAN and transmits the event information quickly. The LAN reports the location information of the registered SRD to the location server at each fixed update period and configures the overall location recognition system; it also periodically communicates with the SRD to check and manage connectivity.

**Figure 7:**
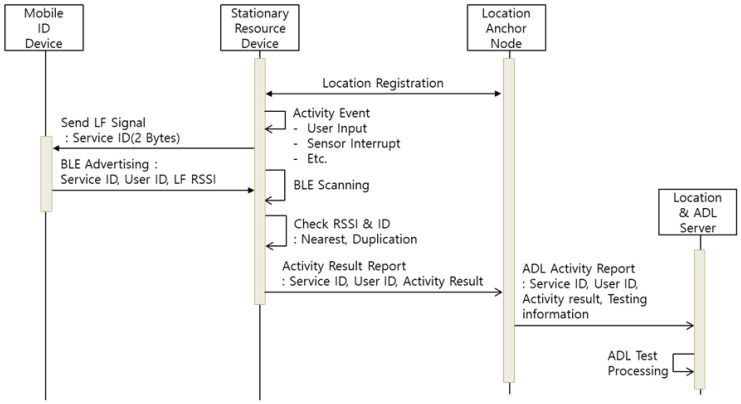
Sequence Diagram of Activity Event Report.

**Figure 8:**
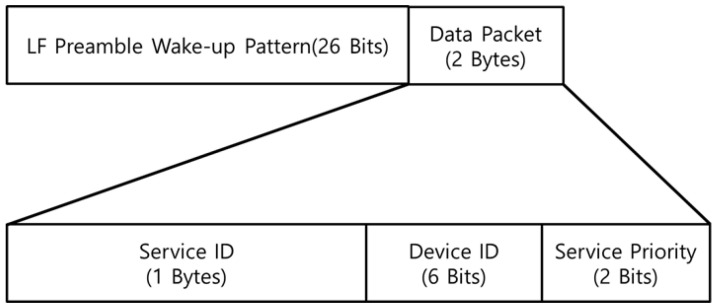
Low frequency (LF) Signal Packet Format.

When a user activity event occurs, the SRD performs user recognition with the procedure shown in Figure 7 and reports the result of the activity event. After the location registration is complete, the SRD recognizes the event through the user/device input, sensor interrupt, etc. Then, the SRD transmits an LF signal containing 2-bytes of service ID information, and the MID around the SRD is activated through the LF signal. Figure 8 shows the packet information of the transmitted LF signal, which includes a unique preamble pattern to activate the MID, and sends the information of the LF signal in 2 bytes of the data packet. The 2-byte LF data packet is composed of the service ID and device ID of the SRD. The service priority is used as a delimiter to classify the LF signals that should be processed first by the MID. The MID puts the service ID and RSSI information of the received LF signal in the BLE advertising and broadcasts it to the surrounding SRD. The SRD then estimates a user (MID) who has generated the activity event by receiving the advertising information of the MID. Subsequently, the SRD reports the ID, result, and user information of the activity event to the corresponding LAN. Finally, the LAN transmits the event report for processing by the ADL server.

#### 4.2.4. Entrance Recognition System

**Figure 9:**
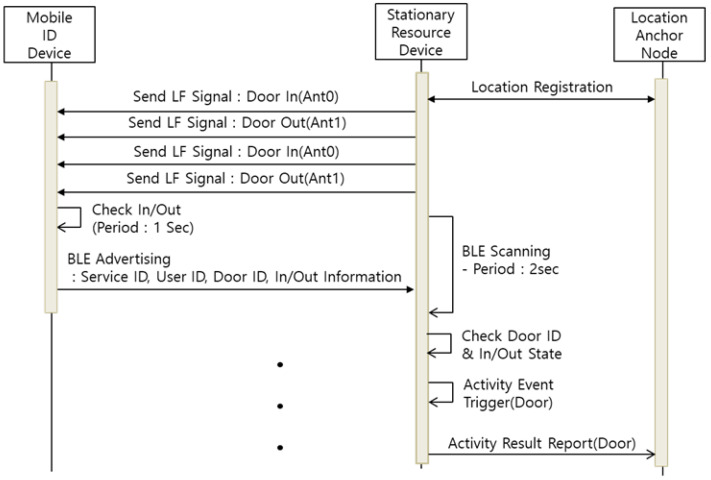
Sequence Diagram of Entrance Recognition System.

In this paper, we propose a user entrance recognition system (ERS) by applying the LF-BLE communication method of the MID and SRD. The ERS transmits two different LF signals to determine whether the MID is within or outside a particular location point. Figure 9 describes the sequence diagram of the ERS.

The SRD transmits two LF signals alternately in a 250 ms cycle. The LF signal includes the ERS service ID, door ID, and access (In/Out) information. The ERS LF antennas are installed inside and outside the entrance. The MID collects LF signals for 1 s and compares the signal strength of the two collected LF signals to determine the current access status. Then, the MID broadcasts its access information through a BLE advertising message; the SRD installed at the door collects the BLE advertising and stores the MID’s access information. When the stored access information is changed, the SRD transmits the activity event to the LAN through the event report. Through the proposed ERS, the movement range record and time of the patient wearing the wearable device are collected on the LAN. It is not possible to replace the gait test performed in a hospital through analysis of the stored movement record. However, the patient’s reduced mobility can be recognized through long-term movement records. In addition, it is expected to be able to analyze various factors such as motion congestion and increased travel time of patients through long-term recorded information.

#### 4.2.5. External Sensor Device

**Figure 10:**
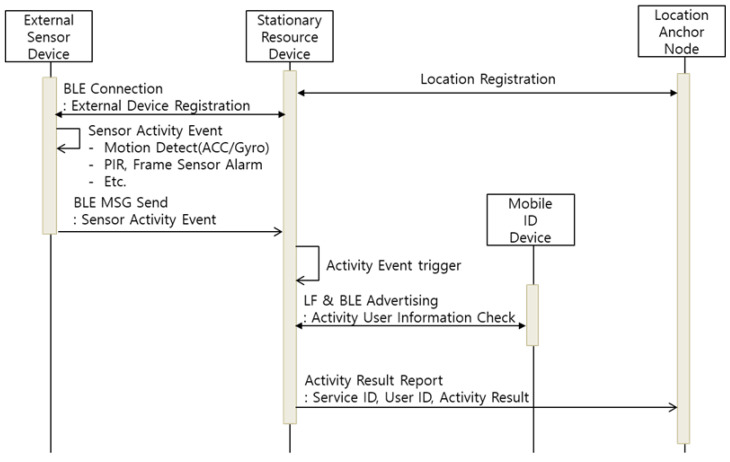
Sequence Diagram of Activity Event Report (External Sensor Device).

The user activity event includes not only the state of use of household appliances, such as TVs and microwaves, but also various life activities such as water use, window opening and closing, and cabinet use. The SRD can be connected to several home appliances to measure activity events, but some activity events need to be measured through external environmental sensors. The ESD includes various external environmental sensors, such as acceleration, temperature/humidity, air quality, and motion sensors. Through this, the ESD can measure physical activity events such as water use, window opening and closing, and environmental information around the users, such as temperature/humidity. The ESD is designed as a mobile device that does not include an LF transmitter or does not have a fixed power supply depending on the installation characteristics. For this reason, the SRD with LF transmission function must be additionally used, if necessary, to process the activity event report. Figure 10 represents the activity event report process through the ESD and SRD.

The ESD recognizes user activity events through built-in sensors. For example, the faucet tag, which is a type of ESD, is installed atop the handle of the faucet and periodically measures the *Y*-axis value of the acceleration sensor to determine whether the faucet is opened or closed through changes in the measured value. The ESD generates the activity event through changing of the faucet’s open–close state and transmits the event occurrence and result to the SRD registered. Then, the SRD performs user recognition by searching the surrounding MID at the time of operation. Finally, the SRD transmits the service ID, user ID, and activity result to the LAN.

**Figure 11:**
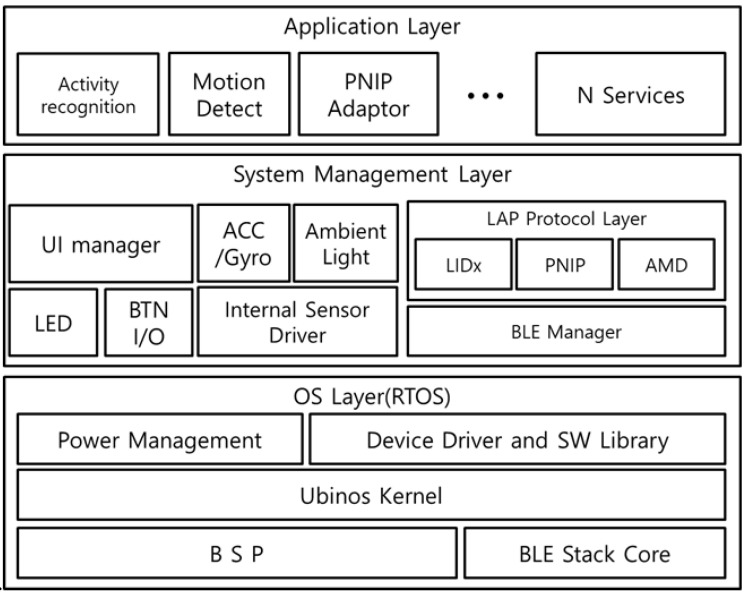
S/W Architecture of External Sensor Device.

Figure 11 shows the S/W architecture of the ESD, which uses the same RTOS-based OS layer as the SRD [41]. The system management layer includes various sensor interfaces of the ESD and communication protocols for the activity event reports. In the application layer, activity recognition and motion detection are performed with the sensor data collected through the internal sensor interface. The connection with the SRD for user identification is managed through the Proximity-based Neighbor Identification Protocol(PNIP) adapter of the application layer. The PNIP adapter automatically performs BLE connection with the matched SRD through the corresponding ESD ID information. In addition, the PNIP adapter recovers any BLE connection disruption due to a communication state error between the ESD and SRD to deliver the user activity events.

## 5. Implementation and Evaluation

### 5.1. Implementation of Smart Diagnosing System

**Figure 12:**
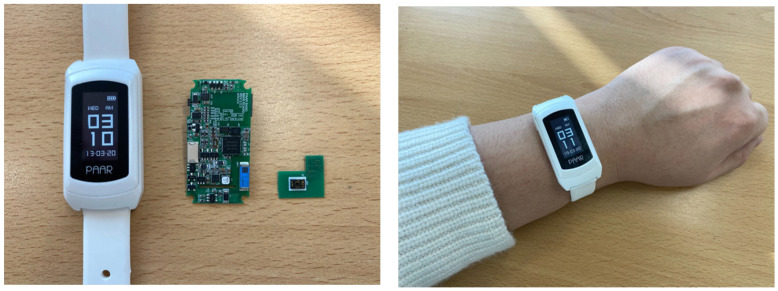
Implementation of Mobile Identification Device (Smart Band Type).

**Figure 13:**
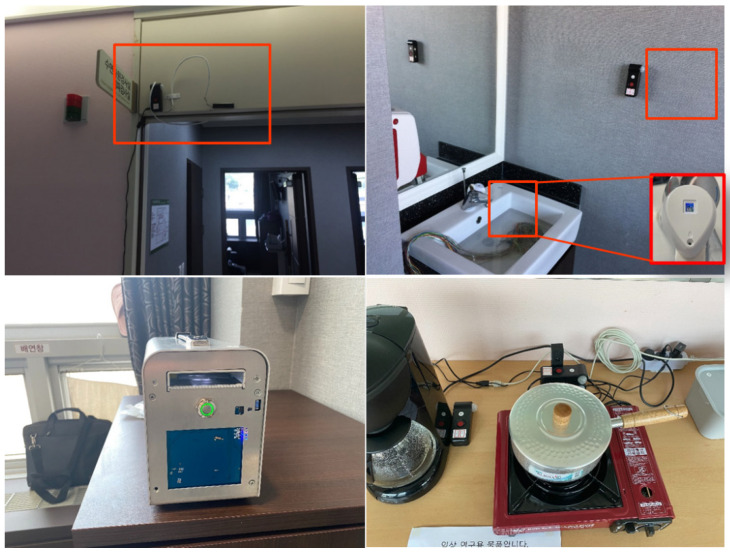
Installation of Resource Device for Pilot Test.

The smart diagnosing system is implemented with the MID, SRD, ESD, and LAN. The MID is designed as a smart band and mobile tag type to make it easy for the users to wear. During the test through the smart diagnosing system, the user wears a smart band, as shown in Figure 12, for user identification of the activity events. For limited resource management of mobile devices, the MID was developed with low-power RTOS. Further, the MID’s LF communication method supports asynchronous M2M communication to optimize power consumption for user identification communication. The SRD provides various digital/analog interfaces, such as SPI, UART, I2C, USB, GPIO, and ADC, through the external sensor interface on the rear. As shown in Figure 13, the SRD detects the user events either when physically connected to the device or through built-in sensors. For example, in the case of the gas stove activity event detection, a flame sensor is connected to the SRD to detect the flame of the gas stove to determine its use. The blood pressure monitors, coffee pots, microwave ovens, etc. that have digital signal interfaces receive the activity events of the corresponding equipment through an interrupt through the GPIO or a wired communication method. In addition, the two SRDs at the room entrance are installed inside and outside, as shown in Figure 12, to detect the movement of the MID.

**Figure 14:**
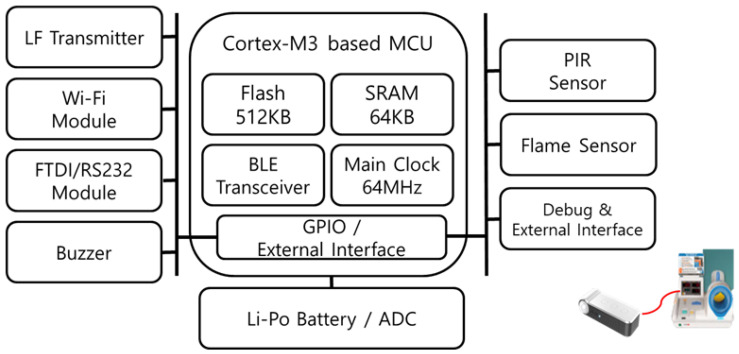
Hardware Block Diagram of Stationary Resource.

Figure 14 describes the hardware block diagram of the SRD, which has an LF transmitter, Wifi module, and BLE transceiver for M2M communication. These external communication modules maintain communication between the IoT device and the central hub. In addition, the SRD consists of an external interface for connection with the home appliances and several built-in sensors, such as PIR and frame sensors.

**Figure 15:**
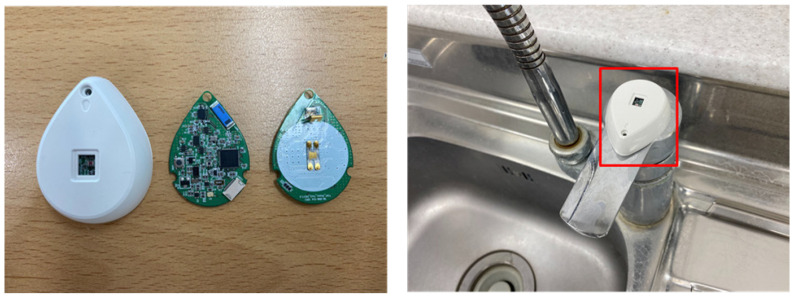
External Sensor Device (Faucet).

The ESD performs various activity recognition tasks through the built-in sensors. Figure 15 shows an ESD that is attached to the faucet with an acceleration sensor to detect opening or closing. This type of ESD has limited resources without a fixed power supply. However, it is easy to install, so it detects physical user activity events, such as movement of drawers, windows, and kitchen utensils, that the SRD cannot recognize. The LAN is installed in each unit cell with a BLE communication area of a maximum of 10–15 m. The LAN also manages devices existing in its own cell through BLE communication and delivers the activity event report collected from these devices to the ADL server through the IP network.

**Figure 16:**
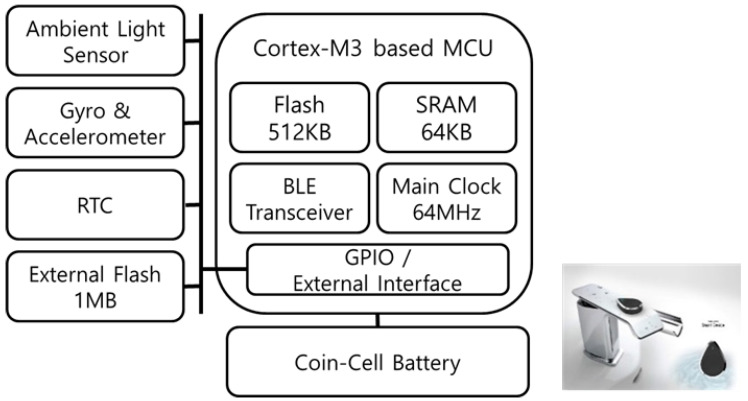
Hardware Block Diagram of External Sensor Device.

The ESD uses the MID’s core hardware structure as is and excludes unnecessary user interfaces by incorporating various environmental and gyro/accelerometer sensors. Further, it is composed of the RTC and an external flash memory for data collection and self-time information management. Figure 16 shows the hardware block diagram of the ESD.

### 5.2. Test Analisys of ADL Diagnosing System

In this study, tests were performed on hospital patients to verify the proposed ADL diagnosing system. The tests were conducted on 38 elderly patients for about a month. The test patient group was classified into 15 normal patients, 11 persons with MCI, and 12 persons with dementia. Each patient performed the movement, toilet use, washing face, and home appliance/medical device use according to the scenarios described in Section II. The results of the ADL events were collected through the SRD, ESD, and LAN installed in each test room. Table 4 shows the average success rate and time required for each test performed by the patients.

### 5.3. Response Time and Power Consumption of ADL Report Protocol

**Figure 17:**
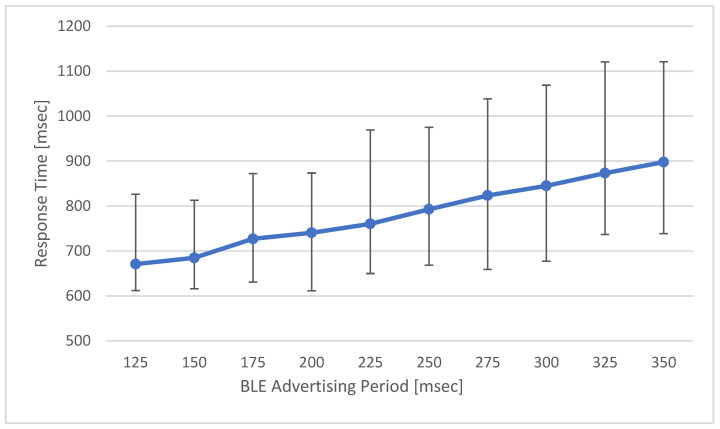
Response Time of ADL Report Protocol.

The ADL report protocol proposed in this work consists of two steps. The first is a user identification step using the BLE advertising information by activating the MID through LF signal transmission. In this step, the BLE scan period of the SRD is set according to the BLE advertising period of the MID that receives the LF signal. The second step is the process of delivering the user information and activity results to the LAN; since this process is transmitted through an already connected BLE channel, it has an almost constant execution time. Therefore, the response time of the entire ADL report protocol is proportional to the BLE advertising period in the user identification stage. In this test, the response time of the ADL report protocol was measured according to changes in the BLE advertising period of the MID. Figure 17 shows these test results.

**Figure 18:**
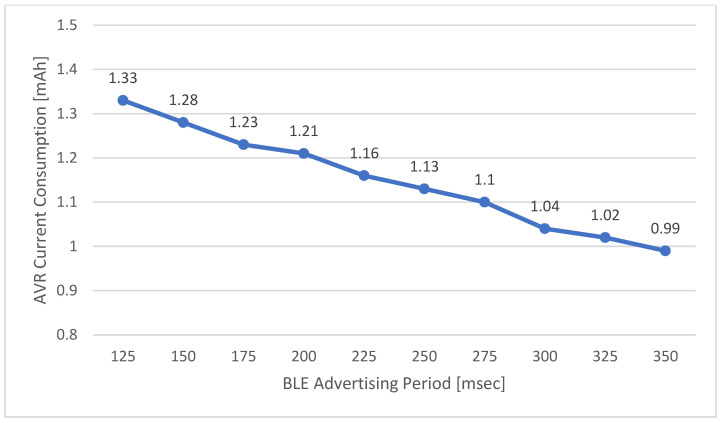
Average Power Consumption of ADL Report Protocol (Mobile Identification Device (MID)).

The response time of the ADL report protocol tends to increase proportionally with the BLE advertising scan time in the user identification process. This scan time decreases as the BLE advertising period of the MID decreases. However, frequent BLE advertising cycles greatly affect the power consumption of the MID. Therefore, the power consumption of the entire ADL report protocol operation according to the MID’s BLE advertising period is shown in Figure 18. In the ADL report protocol, the response time increases as the BLE advertising period of the MID reduces. However, the shorter the BLE advertising period is, the more the power consumption of the MID. The MID is a mobile device using a battery and requires limited power consumption; thus, it is necessary to maintain appropriate response time of the ADL report.

This system not only collects user ADL activity logs, but also considers services such as alarms for user abnormal behavior through the E-coaching system. When performing a long-term analysis, a quick response time for real-time and responsiveness is not required, but for a system that directly helps the patient’s daily life or for safety, the response time must be designed according to the response time required by the system. In this study, in order to satisfy these requirements, long-term collection data are transmitted from LAN to external server, but in the case of data necessary for processing internal events and services, services are implemented only with local LAN, RD, and MID devices. Currently, various services are being built through the implemented platform, and time requirements for the service are being derived.

### 5.4. Response Time of Entrance Recognition 

**Figure 19:**
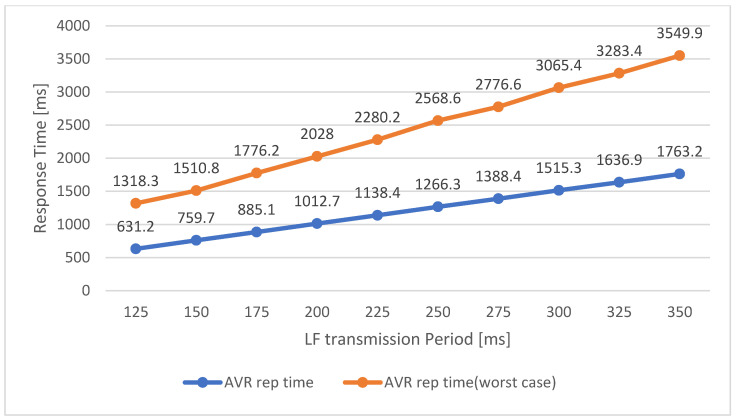
Response Time of Entrance Recognition System.

The ERS transmits the LF signals more frequently than the general ADL report protocol. In addition, the ERS transmits the results to the LAN at the same time as user recognition through BLE advertising of the MID. Since the ERS must complete recognition within the user’s moving time, there is a difference in performance depending on the response time of the service. Moreover, to minimize the response time, the MID sets the LF scan window according to the LF transmission period of the SRD of the ERS. The MID then collects and analyzes the LF information during the LF scan window, after which it transmits the results to the surrounding SRD through the BLE advertising message. The LF scan window of the MID must collect at least two pairs of LF signals from the ERS. Therefore, in the proposed system, the LF transmission period is set to five times for stable operation. Hence, the LF scan window and response time are shortened according to the LF transmission period, and the user’s entrance/exit movement event must be completed within this response time. Therefore, the faster the movement, the shorter is the response time required. Figure 19 describes the results of response time measurements of the ERS according to the LF transmission period.

**Figure 20:**
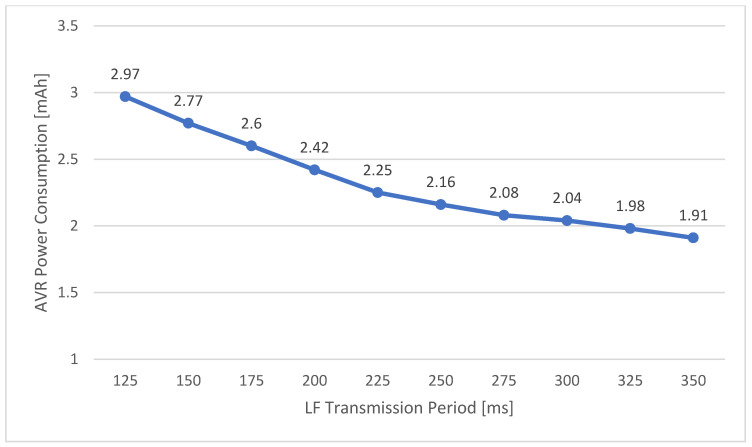
Power Consumption of Entrance Recognition System.

This test analyzed the response time when the access is normally recognized through the first LF scan window, and the worst case was recognized through the second LF scan window. In both cases, the response time is proportional to the LF transmission period. As a result, the LF transmission time of the ERS should be designed so that the user’s moving time can be obtained in the worst-case scenario. Because the SRD that transmits the LF signal generally has a fixed power supply, the power consumption is not limited. However, the MID that is activated and operated after receiving the LF signal through the access system is limited by the power consumption. The period of the LF signal transmitted through the ERS affects the power consumption of this MID, and the power consumption varies according to the corresponding period, as shown in Figure 20. The power consumption of the MID is inversely proportional to the LF transmission period, as shown in the graph above, and the power consumption increases as the signal is transmitted over shorter periods. Therefore, even if the LF communication period of the ERS satisfies the user’s movement speed, the LF transmission period of the slowest possible period should be set considering the limited power consumption of the MID.

## 6. Conclusions

In this paper, we proposed a system to recognize ADL through self-organized M2M communication technology of IoT sensor devices and a sensor network. The proposed system is designed with an RD, an MID, and a LAN, which are the IoT devices used to recognize ADL events of patients. The RD was designed as SRD and ESD according to the recognition method. The SRD is connected to several sensors and home appliances to detect user ADL events. The ESD directly detects actions, such as washing hands and opening doors, through built-in sensors such as accelerometers. In addition, to recognize ADL more efficiently, the RD and MID were developed by mixing LF communication for activation and distance recognition functions with BLE for data communication. In this work, we designed the ADL report protocol for self-organized M2M communication between devices using these two communication methods. Finally, a LAN was implemented to manage and operate the RD and MID within its own cell and to transmit the collected ADL to the server. Using these ADL collection devices, we installed IoT sensor devices and designed pilot test scenarios to implement/verify the diagnostic system. For each test step, the ADL events were collected with the proposed devices to ascertain the patient’s mobility, tool use ability, and cognitive ability. The tests were controlled through the E-coaching system. As a verification of the pilot test, the response time of the activity report protocol, power consumption, and performance of the entrance system for movement detection were analyzed. In this study, a pilot test for verification of the proposed system was conducted.

The ADL measurement system proposed in this paper has practical limitations to be used as a medical device. In order to register medical device equipment, a clear medical device classification is required for the device, and there are high-level requirements for accuracy, reliability, and functionality. Currently, there is no clear standard for the user’s activity pattern recorded through ADL measurement to be used as medical information. In addition, the ADL data measured through the sensor are insufficient for accurate diagnosis of disease. Therefore, the proposed system aims to use a basic test tool to analyze the user’s life pattern before applying the medical diagnosis level of the hospital. When diagnosing diseases such as MCI and dementia, long-term analysis of life patterns measured through the proposed system can be used as support information for diagnosis. In the future, we will study the standardization of life pattern information through cooperation with hospital experts. In future research, this system can be installed directly at home and used to collect and analyze ADL events in real-life scenarios. In the future, this system will apply ADL recognition and the E-coaching system through the SMART home concept. The collected data can be used to analyze the symptoms of brain disease by classifying the patient’s daily life patterns. Furthermore, through this system, a system for life safety is planned to be developed by detecting abnormal behaviors of patients in real-time environments and by broadcasting alarms and stopping usage of home appliances accordingly.

## Figures and Tables

**Table 1:** ADL Test Configuration and Sequence.

Test Room 1	Test Room 2
1. Test start (button input) 2. Enter Test Room 1 3. Enter toilet 4. Use toilet 5. Discard after using paper towel 6. Exit toilet 7. Use washstand 8. Use sphygmomanometer 9. Take medicine (smart pill reminder) 10. Exit Test Room 1	11. Enter Test Room 2 12. Use Gas Stove 13. Use Coffee port 14. Use Microwave 15. Exit Test Room 2 16. Enter Test Room 1 17. Test End (Button Input)

**Table 2:** Service ID for ADL Events.

Service Name	Service ID(Hex)	Service Type	Service Name	Service ID(Hex)	Service Type
Blood Pressure	0x01	Resource	Body Fat Monitor	0x0D	Resource
Weight	0x02	Resource	Vital Signal Alarm	0x0E	Resource
Blood Sugar	0x03	Resource	SPO2	0x0F	Resource
Medication	0x04	Resource	Entrance	0x10	Resource
Door Lock	0x05	Resource	TAP Water	0x11	Resource
Gas Stove	0x06	Resource	Gas Tag	0x12	Resource
Fitness Equipment	0x07	Resource	Smart Toilet	0x13	Resource
Light Control	0x08	Resource	Smart Band	0x14	Mobile
Notification	0x09	Location Service	Smart Tag	0x15	Mobile
Business Card	0x0A	Resource	Environment Sensor	0x16	Resource
Coffee Port	0x0B	Resource	433 MHz Sensor	0x17	Resource
Pedometer	0x0C	Resource	Fitness Prescription	0x18	Resource

**Table 3:** Bluetooth Low Energy (BLE) Advertising Data for ADL Diagnosing System.

Index	Value(Hex)	Description
0	0x02	EAS Field Length
1	0x01	GAP_ADTYPE_FLAGS
2	0x06	GAP_ADTYPE_FLAGS_GENERAL(0x02) + GAP_ADTYPE_FLAGS_BREDR_NOT_SUPPORTED
3	0x13	EAS Field Length
4	0xFF	GAP_ADTYPE_MANUFACTURER_SPECIFIC
5	0x0D	MANUFACTURER ID0
6	0x00	MANUFACTURER ID1
7	0x00	Status Byte (0x00:Idle, 0x01: Identification, 0x02: For SMART Phone, 0x08: Location Request & Registration)
8	0xXX	Device ID0
9	0xXX	Device ID1
10	0xXX	Device ID2
11	0xXX	Device ID3
12	0xXX	LF RSSI Value(0x00 ~ 0xFF dBm)
13	0xXX	LAN MAC Address0 (Location Registration)
14	0xXX	LAN MAC Address1 (Location Registration)
15	0xXX	LAN MAC Address2 (Location Registration)
16	0xXX	LAN MAC Address3 (Location Registration)
17	0xXX	LAN MAC Address4 (Location Registration)
18	0xXX	LAN MAC Address5 (Location Registration)
19	0x15	Packet Tx Profile Handle 0
20	0x00	Packet Tx Profile Handle 1
21	0x14	Packet Rx Profile Handle 0
22	0x00	Packet Rx Profile Handle 1
23	0x13	Packet Tx CCCD Handle 0
24	0x00	Packet Tx CCCD Handle 1

**Table 4:** Test Result of ADL Diagnosing System.

ADL Event	Activity Type	Success Ratio (%)			Success Time (s)		
		Normal	MCI	Dementia	Normal	MCI	Dementia
Movement	Passage -> Room 1	100	100	100	5.87	8.97	11.52
Movement	Room 1 -> Bathroom	100	90.91	75	10.67	11.96	12.90
Tool using	Toilet	86.67	81.81	83.33	31.60	39.46	60.44
Movement	Bathroom ->handbasin	100	81.82	83.33	11.35	10.01	15.54
Tool using	Hand washing	93.33	100	41.67	20.75	29.44	25.78
Tool using	Hand wiping	100	90.91	41.67	20.15	18.60	24.84
Tool using	Sphygmomanometer	100	90.91	66.67	76.63	85.04	107.77
Tool using	Pill reminder	93.33	45.45	25.00	100.57	102.26	86.55
Movement	Room ->Dining room	100	72.73	16.67	35.27	88.06	90.10
Tool using	Gas stove	93.33	54.55	25	52.99	50.09	77.68
Tool using	Coffee machine	100	81.82	50	38.69	49.17	74.08
Tool using	Microwave	100.00	54.55	0.00	58.81	72.27	180.00
Movement	Dining room -> Room	100	81.82	50	49.98	69.28	180.00
